# Red blood cell transfusion in patients with ST-elevation myocardial infarction—a meta-analysis of more than 21,000 patients

**DOI:** 10.1007/s12471-018-1137-x

**Published:** 2018-07-23

**Authors:** R. I. Mincu, T. Rassaf, M. Totzeck

**Affiliations:** 1 Medical Faculty, West German Heart and Vascular Center, Department of Cardiology and Vascular Medicine, University Hospital Essen, Essen, Germany; 2 University and Emergency Hospital, Cardiac Research Unit, ‘Carol Davila’ University of Medicine and Pharmacy, Bucharest, Romania

**Keywords:** Red blood cell transfusion, ST-elevation myocardial infarction, Meta-analysis, Mortality rate, Reinfarction rate

## Abstract

**Background:**

Red blood cell transfusion remains controversial in patients with acute coronary syndromes and particularly in patients with ST-elevation myocardial infarction (STEMI).

**Methods:**

We systematically searched PubMed, Cochrane, EMBASE, and Web of Science for studies published until January 2017 describing the outcomes in patients with STEMI who received red blood cell transfusion, compared with patients who did not.

**Results:**

A total of 21,770 patients with STEMI from 5 cohort studies were included in the meta-analysis, 984 (4.5%) received red blood cell transfusion and 20,786 (95.4%) did not. Red blood cell transfusion was associated with a higher risk of in-hospital and long-term mortality, emergency repeated percutaneous coronary intervention (PCI), reinfarction rate, stroke rate, and heart failure. The group with red blood cell transfusion had a slightly higher incidence of diabetes mellitus and hypertension, but a lower incidence of smoking. The two groups had the same incidence of prior myocardial infarction, prior coronary artery bypass graft surgery and malignancy. Prior heart failure, prior stroke and prior PCI were more frequent in the group that had received red blood cell transfusion. The mean nadir haemoglobin was 8.5 ± 0.1 g/dl in the group with red blood cell transfusion and 12.5 ± 0.4 g/dl in the control group, *p* < 0.001.

**Conclusions:**

Red blood cell transfusion increases the morbidity and mortality in patients with STEMI. This difference could not be explained by the higher morbidity in the red blood cell transfusion group alone. Further randomised controlled trials are required to provide a reliable haemoglobin threshold for these patients.

**Electronic supplementary material:**

The online version of this article (10.1007/s12471-018-1137-x) contains supplementary material, which is available to authorized users.

## What’s new?

- The outcomes for patients with acute coronary syndromes receiving red blood cell transfusion are not fully characterised.
- Red blood cell transfusion is associated with higher in-hospital and long-term mortality in STEMI patients.
- Repeated percutaneous coronary intervention is more frequent in patients following transfusion.

## Introduction

The pros and cons of red blood cell transfusion (RBT) during acute coronary syndromes are controversial. There is a paradox between anaemia necessitating administration of RBT in acute coronary syndromes and the inferior outcomes after RBT reported by several studies [1–4].

Acute coronary syndromes describe the range of myocardial ischaemic states that include: unstable angina, defined as partially or intermittent coronary artery occlusion without myocardial injury, non-ST-elevation myocardial infarction (NSTEMI), defined as partially or intermittent coronary artery occlusion with myocardial damage, and ST-elevation myocardial infarction (STEMI), defined as complete coronary artery occlusion with myocardial damage [5, 6].

Several studies have demonstrated a strong association between RBT and mortality and morbidity in the setting of all types of acute coronary syndromes, but the outcomes in patients with STEMI are still not fully described and understood [2, 4, 7–10].

The lack of specific guideline indications for RBT in patients with STEMI, the absence of well-designed randomised controlled trials (RCTs) as well as the concurrent effects of anaemia, acute bleeding events and other comorbidities on the outcomes of this population, make a complete definition and description of causes and magnitude of adverse effects challenging. We conducted a meta-analysis to determine the impact of RBT on short-term and long-term outcomes in patients with STEMI, in order to address the gaps in knowledge in the management of these patients.

## Methods

The methods used to perform this work were in compliance with the MOOSE (Meta-analysis Of Observational Studies in Epidemiology) group recommendations [11]. We used the PRISMA (Preferred Reporting of Items for Systematic Meta-Analysis) algorithm for study selection [12]. The methodology has been reported before and it is detailed in the online supplementary material [13].

## Results

### Study selection

**Fig. 1:**
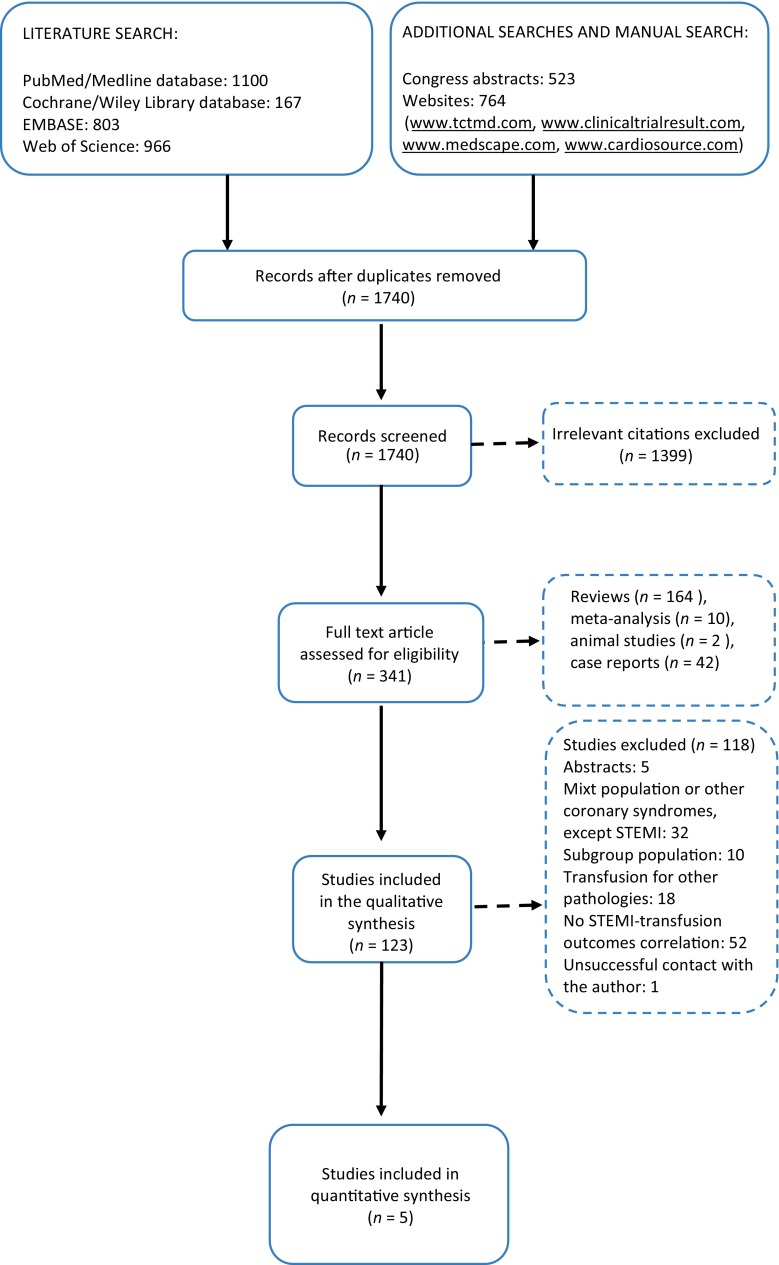
PRISMA flow chart for the study selection [12]

We selected 5 cohort studies to be included in our meta-analysis [4, 14–17]. The study selection process is shown in Fig. 1. Overall, there were 21,770 patients included in our analysis, 984 in the RBT group and 20,786 in the control group. The follow-up period varied between 3 and 60 months. The characteristics of the selected studies are shown in the online supplementary Table 1. The quality of the included studies was high, according to the Newcastle-Ottawa Scale (online supplementary Table 2).

The incidence of cardiovascular risk factors between groups is presented in the online supplementary Table 1. Diabetes mellitus was slightly higher in the RBT group (relative risk [RR] = 1.39; 95% CI 1.24–1.56; *p* < 0.001), as well as the incidence of arterial hypertension (RR = 1.2; 95% CI 1.11–1.3; *p* < 0.001). The incidence of smoking was lower in the RBT group compared with controls (RR = 0.77; 95% CI 0.62–0.96; *p* = 0.02). However, the overall profile of cardiovascular risk factors was not different between groups (RR = 1.11; 95% CI 0.99–1.06; *p* = 0.09).

The two groups had the same incidence of prior myocardial infarction (RR = 1.29; 95% CI 0.89–1.88; *p* = 0.18) prior coronary artery bypass graft (RR = 1.43; 95% CI 0.98–2.09; *p* = 0.06) and malignancy (RR = 0.99; 95% CI 0.75–1.30; *p* = 0.95). The following pathologies were more frequent in the RBT group: prior heart failure (RR = 1.76; 95% CI 1.24–2.48; *p* < 0.001), prior stroke (RR = 1.76; 95% CI 1.24–2.48; *p* = 0.001), and prior PCI (RR = 1.33; 95% CI 1–1.76; *p* = 0.05). The overall profile of the medical history showed a significantly higher incidence of morbidity in the RBT group (RR = 1.36; 95% CI 1.18–1.56; *p* < 0.001).

The mean nadir haemoglobin was 8.5 ± 0.1 g/dl in the RBT group and 12.5 ± 0.4 g/dl in the control group, *p* < 0.001, as reported in three studies [4, 14, 16]. The haemoglobin threshold for administration of RBT was not specified in the studies. One study [14] indicated that 46.7% of RBT were administered in the absence of moderate or severe bleeding events, whereas one study [4] indicated that 81.7% of the patients who received RBT had a moderate or major bleeding event. The incidence of moderate or major bleeding events among the RBT group, was reported by three studies [4, 14, 17] as being 81.7%, 53.4% and 97%, respectively. The incidence of anaemia at baseline was reported by one study [14] to be 87% in the RBT group and 23% in the control group.

### RBT and in-hospital mortality

**Fig. 2:**
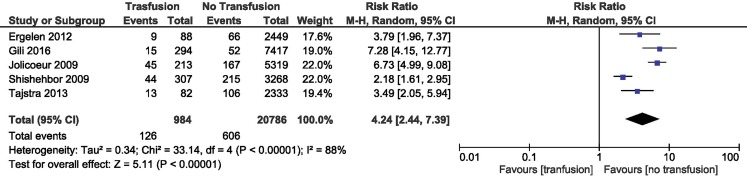
Overall estimate and estimates from each study of the RR for in-hospital mortality associated with RBT. The first author and the publication year were used for each study. The total number of events and the sample size are shown for each study. The weight of each study in the final analysis is indicated as a percentage. The RR for each study is shown numerically on the left and graphically on the right. Square boxes denote the RR, horizontal lines represent 95% confidence intervals, and the diamond plot represents the overall results of the included trials. Weights are from a random effects analysis. *RR* relative risk, *RBT* red blood cell transfusion

RBT was associated with increased in-hospital mortality (RR = 4.24; 95% CI 2.44–7.39; *p* < 0.001). The result was pooled from 5 studies [4, 14–17] with a total number of 21,770 patients, 984 in the RBT group and 20,786 in the control group. The heterogeneity between the studies was significant (Fig. 2).

### RBT and long-term mortality

**Fig. 3:**
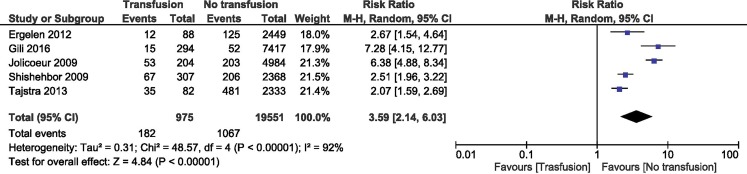
Overall estimate and estimates from each study of the RR for long-term mortality associated with RBT. The first author and the publication year were used for each study. The total number of events and the sample size are shown for each study. The weight of each study in the final analysis is indicated as a percentage. The RR for each study is shown numerically on the left and graphically on the right. Square boxes denote the RR, horizontal lines represent 95% confidence intervals, and the diamond plot represents the overall results of the included trials. Weights are from a random effects analysis. *RR* relative risk, *RBT* red blood cell transfusion

Patients with STEMI who received RBT had a higher risk of mortality (RR = 3.59; 95% CI 2.14–6.03; *p* < 0.001) compared with the control population. We analysed data from 20,526 patients divided in two groups: 975 in the RBT group and 19,551 in the control group. The heterogeneity between studies was significant (Fig. 3).

### RBT and reinfarction rate

**Fig. 4:**
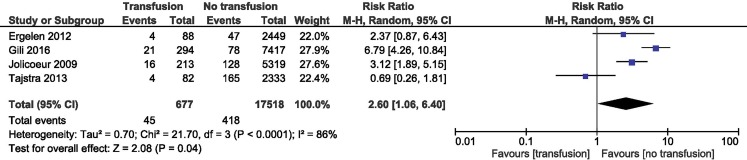
Overall estimate and estimates from each study of the RR for reinfarction rate associated with RBT. The first author and the publication year were used for each study. The total number of events and the sample size are shown for each study. The weight of each study in the final analysis is indicated as a percentage. The RR for each study is shown numerically on the left and graphically on the right. Square boxes denote the RR, horizontal lines represent 95% confidence intervals, and the diamond plot represents the overall results of the included trials. Weights are from random effects analysis. *RR* relative risk, *RBT* red blood cell transfusion

RBT was associated with a higher risk of reinfarction compared with controls (RR = 2.60; 95% CI 1.06–6.4; *p* = 0.04). We summarised data from 18,195 patients with STEMI: 677 in the RBT group and 17,518 in the control group. The heterogeneity between studies was significant (Fig. 4).

### RBT and emergency repeated PCI

Patients with STEMI who received RBT were at a higher risk of emergency repeated PCI compared with patients who did not receive RBT (RR = 1.4; 95% CI 1.01–1.95; *p* = 0.05). There were 383 patients in the RBT group and 10,101 in the control group. The heterogeneity between studies was significant.

### RBT and stroke rate

The stroke rate was higher for patients with STEMI who received RBT compared with patients who did not receive RBT (RR = 3.26; 95% CI 1.20–8.85; *p* = 0.02). The analysis pooled data from 7719 patients: 286 patients in the RBT group and 7433 in the control group. The heterogeneity between group was not significant.

### RBT and heart failure

Patients with STEMI who received RBT were at higher risk of developing heart failure compared to the control population (RR = 2.67; 95% CI 1.43–5.00; *p* = 0.002). The data was extracted from 8069 patients: 301 patients in the RBT group and 7768 in the control group. The heterogeneity between studies was significant.

### RBT and drug-eluting stent implantation for the culprit lesion

Patients with STEMI from the RBT group (984 patients) and from the control group (20,786 patients) were treated equally with drug-eluting stents (RR = 0.95; 95% CI 0.84–1.08; *p* = 0.45). The heterogeneity between studies was significant.

### Heterogeneity between studies, inconsistency and publication bias

The heterogeneity and inconsistency between studies were significant, as described above. The quality of the included studies was high, as reflected by the result of the Newcastle-Ottawa Scale (Supplementary Table 2).

### The sensitivity analysis

A sensitivity analysis was performed to address the relative importance of each study, by excluding each study in turn from the analysis. The predictive value of the RBT is valid for all outcomes, except for the emergency repeated PCI and stroke, where removing the data from one study [14], makes the comparison statistically insignificant.

### The subgroup analysis

The mean age of the RBT group was 67 ± 3 years and the mean age of the control group was 61 ± 4 years (*p* = 0.02). There were less males in the RBT group, compared with controls (49.2 ± 7.8% vs. 78.2 ± 3.8%, *p* < 0.001).

## Discussion

We performed a meta-analysis to determine the impact of RBT on short-term and long-term outcomes for patients with STEMI. The main findings of our study were: (a) patients with STEMI who received RBT had a higher risk of in-hospital and long-term mortality compared with STEMI patients who did not receive transfusion, (b) the reinfarction rates and the rates of emergency repeated PCI were higher in the RBT group compared with controls, (c) the stroke and heart failure risks were higher in the RBT group compared with controls, (d) patients who received RBT were older and predominantly female, (e) patients with STEMI who received RBT had a higher incidence of hypertension and diabetes mellitus, but a lower incidence of smoking, as well as a higher incidence of prior heart failure, prior stroke and prior PCI, but an equal incidence of prior myocardial infarction, prior coronary artery bypass graft and prior carcinoma compared with the control population, and (f) the mean nadir haemoglobin in the RBT group was 8.5 ± 0.1 g/dl.

When taken separately, the differences in age, sex and morbidity between the two groups may seem sufficient if we want to explain the differences in outcomes. However, the results are consistently significant and suggest that RBT is a risk factor for STEMI patients that should not be underestimated. It requires further research and needs to be understood to reduce the morbidity in this population.

The mechanisms through which RBT could lead to pathological outcomes in patients with STEMI have not been completely elucidated. One hypothesis would be that transfused red blood cells suffer from a ‘storage lesion’ that could affect their ability to deliver oxygen to the tissues and are depleted of nitric oxide that would not allow them to appropriately interact with the endothelium and deliver oxygen to the ischaemic tissues [18]. Other hypotheses would be that RBT solutions contain prothrombotic activators that generate platelet activation or that RBT would decrease microcirculatory flow in patients with STEMI [19, 20]. Additionally, the withdrawal of survival prolonging medication in the setting of STEMI due to bleeding events could increase the risk of negative outcomes [3].

Studies that assessed the balance between the risks and benefits of RBT are contradictory. There are studies that reported beneficial or neutral effect of RBT for patients with acute coronary syndromes at haemoglobin levels below 8 g/dl, and harmful effects if RBT was undertaken at haemoglobin levels above 11 g/dl [21]. A meta-analysis suggested that a liberal threshold of more than 8 g/dl would be safer in patients with ongoing acute coronary syndrome or chronic cardiovascular disease, until high quality randomised trials are available [22]. One article reports a threshold of 12 g/dl haemoglobin to be associated with a decreased risk of cardiovascular death among patients with STEMI [23]. One meta-analysis performed on STEMI and NSTEMI patients who received RBT, reported a higher risk of mortality and recurrence of myocardial infarction in these patients compared with patients who did not receive RBT, which is similar with our findings. However, when pooling data for STEMI alone, the outcomes were similar in both groups [7]. Overall, studies that included patients with major bleeding events in the analysis showed an important impact of RBT on outcomes [9, 24], whereas the studies that excluded patients with bleeding events were neutral or inconclusive [23, 25, 26].

European and American guidelines for the management of RBT do not provide clear indications for RBT in the setting of STEMI, due to incomplete evidence [27, 28]. There was a marked variation in transfusion patterns among the STEMI cohort that we have analysed: the threshold for administration of RBT was not specified and there were patients who received RBT in the absence of moderate or severe bleeding events.

The lack of randomisation, the unequal number of cases and controls, the differences in baseline morbidity among groups and the overall low frequency of transfusion (4.5% of all 21,970 patients) impair an accurate conclusion regarding the causal factors of the adverse events described above. Anaemia brings a higher risk of mortality and morbidity per se, thus creating an association between transfusion and outcomes that might not be causal [29]. The available data raise the suspicion of increased morbidity and mortality among patients with STEMI who receive RBT, but the question whether or not the relationship is causal remains unanswered, because we cannot completely eliminate the different factors and pathologies contributing to this phenomenon. That is why almost all analyses of transfusion indication in the setting of STEMI emphasise the imperative need of prospective, well-designed and high quality RCTs, before making a statement that can be safely used in clinical practice [28].

Our study has some limitations that need to be addressed. First, the data provided are from observational trials, not from randomised controlled trials, because there are no randomised controlled trials that meet the inclusion criteria. This could increase the risk of bias. Nevertheless, the quality of the included studies was high. Second, a threshold for the haemoglobin level could not be identified from the available data. Third, we could not accurately identify the immediate causal factors of the outcomes because the patients in the RBT group were older, predominantly female and had comorbidities. However, the groups do reflect clinical reality and this issue could be solved by developing RCTs. Forth, the patients who received RBT had an overall higher risk profile and this may partially explain the outcomes. Fifth, we did not have patient specific data from the selected published studies.

## Conclusion

Patients with STEMI who received RBT have a higher risk of short-term and long-term mortality compared with patients with STEMI who did not receive RBT. They have higher reinfarction and repeated emergency PCI rates, as well as a higher relative risk of stroke and heart failure. Furthermore, they had a similar rate of drug-eluting stent implantation compared with controls. Further RCTs are imperative to address the gaps of evidence in the management of these high-risk patients.

## Electronic Supplementary Material

Information regarding the search strategy, statistical analysis and study end points can be found in the data supplement
