# Supplementary material for: Red blood cell transfusion in patients with ST-elevation myocardial infarction—a meta-analysis of more than 21,000 patients
Source: Neth Heart J. 2018 Jul 23;26(9):454–60. doi: 10.1007/s12471-018-1137-x (PMC6115305; doi:10.1007/s12471-018-1137-x)
Supplement: Supplementary file 1 — Information regarding the search strategy, statistical analysis and study end points can be found in the data supplement [file 12471_2018_1137_MOESM1_ESM.docx]

**Red blood cell transfusion in patients with ST-elevation myocardial infarction**

**- a meta-analysis of more than 21,000 patients**

R. I. Mincu^1,2^, T. Rassaf^1^, and M. Totzeck^1*^

^1^University Hospital Essen, Medical Faculty, West German Heart and Vascular Center, Department of Cardiology and Vascular Medicine, Essen, Germany

^2^ “Carol Davila” University of Medicine and Pharmacy - University and Emergency Hospital, Cardiac Research Unit, Bucharest, Romania

*Corresponding author

Matthias Totzeck

University Hospital Essen

West German Heart and Vascular Center

Clinic for Cardiology and Vascular Medicine

Email: Matthias.Totzeck@uk-essen.de

**Financial support**

RIM was supported by the European Society of Cardiology through a Research Grant (R-2016-013).

*Methods*

*Information sources and search strategies*

A systematic search of studies published until January 2017 was performed through PubMed, Cochrane, EMBASE, and Web of Science databases, through the major cardiology websites (www.tctmd.com, www.clinicaltrialresult.com, www.medscape.com, www.cardiosource.com), and through the abstracts or presentations of annual meetings of the major cardiovascular societies (European Society of Cardiology and its branches, American Heart Association, American College of Cardiology, Society of Cardiovascular Angiography and Intervention, Transcatheter Cardiovascular Therapeutics, and China Interventional Therapeutics).

We made our search specific and sensitive using Medical Subject Headings terms and free text. We considered studies in any language. Supplementary Table 3 describes the search result trough Medline performed on the 2^nd^ of February 2017.

*Inclusion criteria*

Studies that fulfilled all the criteria below were included:

1. RCTs, prospective or retrospective observational design studies.
2. Studies that reported outcomes in patients with STEMI assigned to two groups: patients that received RBT during hospitalisation (RBT group) and patients that did not received BT during hospitalisation (Control group).
3. Sample size > 50 patients.
4. Minimum 30 days of follow-up.

*Exclusion criteria:*

1. Subgroup studies, review studies, animal studies, laboratory studies, abstracts.
2. Mixed populations without reported outcomes in the STEMI subgroup.
3. No relation between RBT and clinical outcomes.

*Data extraction and quality assessment*

Two of the authors (RIM and MT) independently performed data extraction, using a standard data extraction form that contained publication details (name of the first author, year of publication), study design, characteristics of the studied population (sample size, gender distribution, baseline characteristics), and outcomes.

Two of the authors (RIM and MT) assessed independently the trial eligibility, the trail quality, and extracted the data. We contacted per email the corresponding author of one potentially eligible study in order to obtain the exact mortality rates and other outcomes data, but the attempt was unsuccessful. The trial quality was assessed using the Newcastle-Ottawa Scale (1), because the Cochrane Handbook (2) risk of bias refers especially to randomized trials. According to this scale, each study is judged on eight items, categorized into three groups: the selection of the study groups, the comparability of the groups, and the ascertainment of either the exposure or outcome of interest for case-control or cohort studies respectively. A maximum of 4 stars for selection, 2 stars for comparability, and 3 stars for outcomes could be awarded. Stars are awarded such that the highest quality studies are awarded up to 9 stars. The guidelines for reporting the meta-analysis of observational studies recognizes that the use of quality scoring in meta-analysis of observational studies is controversial and recommends the reporting of quality scoring, if it has been done, and subgroup or sensitivity analysis, rather than using the quality scores (3).

*Study endpoints*

The endpoints were: in-hospital and long-term mortality, reinfarction rate, emergency repeated PCI, incidence of stroke and heart failure (HF).

*Statistical analysis*

The meta-analysis was conducted for eligible studies as per risk estimates by two groups: RBT group, that included the patients with STEMI that received RBT and control group, that included the patients with STEMI that did not receive transfusion. Data are expressed as RR and 95% confidence interval (95% CI) for dichotomous outcomes.(4) A random-effect, rather than a fixed-effect was adopted, because this is likely the most appropriate and conservative, accounting for differences among trials. Heterogeneity between studies was assessed by Q statistic and inconsistency was quantified with the I^2^ statistic. Because this test has a poor power in the event of few studies, we considered both the presence of significant heterogeneity at the 10% level of significance and value of I^2^ ≥ 56% as an indicator of significant heterogeneity (5). The presence of publication bias was assessed by Egger´s test.(6) All analyses were conducted using Review Manager version 5.3 (Revman, The Cochrane Collaboration, Oxford, United Kingdom).

References

1. Wells GA, Shea B, O’Connell D, Peterson J, Welch V, Losos M. The Newcastle-Ottawa Scale (NOS) for assessing the quality if nonrandomized studies in meta-analyses [Available from: http://www.ohri.ca/programs/clinical_epidemiology/oxford.htm

2. Higgins JPT, Green D. Cochrane Handbook for Systematic Reviews of Interventions: The Cochrane Collaboration; 2011. Available from: www.handbook.cochrane.org.

3. Stroup DF, Berlin JA, Morton SC, Olkin I, Williamson GD, Rennie D, et al. Meta-analysis of observational studies in epidemiology: a proposal for reporting. Meta-analysis Of Observational Studies in Epidemiology (MOOSE) group. JAMA. 2000;283(15):2008-12.

4. Viera AJ. Odds ratios and risk ratios: what's the difference and why does it matter? South Med J. 2008;101(7):730-4.

5. Higgins JP, Thompson SG. Quantifying heterogeneity in a meta-analysis. Stat Med. 2002;21(11):1539-58.

6. Egger M, Davey Smith G, Schneider M, Minder C. Bias in meta-analysis detected by a simple, graphical test. BMJ. 1997;315(7109):629-34.

7. Shishehbor MH, Madhwal S, Rajagopal V, Hsu A, Kelly P, Gurm HS, et al. Impact of blood transfusion on short- and long-term mortality in patients with ST-segment elevation myocardial infarction. JACC Cardiovasc Interv. 2009;2(1):46-53.

**Tables**

Supplementary Table 1. Characteristics of the studies included in the meta-analysis.

| First author & year | Ergelen 2012 (20) | | | Gili 2016 (21) | | | Jolicoeur 2009 (4) | | | Shishehbor 2009 (22) | | | Tajstra 2013 (23) | | |
| --- | --- | --- | --- | --- | --- | --- | --- | --- | --- | --- | --- | --- | --- | --- | --- |
| Arms | RBT^a^ | No RBT | P value | RBT | No RBT | P value | RBT | No RBT | P value | RBT | No RBT | P value | RBT | No RBT | P value |
| Total patients (% males) | 88 (55) | 2,449 (84) |  | 294 (46) | 7,417 (78) |  | 213 (47) | 5,319 (78) |  | 307 (59) | 3,268 (78) |  | 82 (39) | 2,333 (73) |  |
| Mean age (years ) | 63.6 | 56.2 | < 0.001 | 70.7 | 62.3 | <0.001 | 71 | 61 | 0.001 | 67 | 62 | <0.001 | 67 | 59 | <0.0001 |
| Moderate or severe bleeding (%) | 53.4 | NR^b^ | NR | NR | NR | NR | 81.7 | NR | NR | 97 | 0.6 | < 0.001 | NR | NR | NR |
| Anaemia at admission (%) | 87 | 23 | <0.001 | NR | NR | NR | NR | NR |  | NR | NR | NR | NR | NR | NR |
| Diabetes mellitus (%) | 30.7 | 23.9 | NS | 27.2 | 19.4 | 0.001 | 23 | 15 | 0.007 | 19 | 15 | 0.09 | 30.5 | 19.8 | 0.018 |
| Arterial hypertension (%) | 52.3 | 40.7 | 0.04 | 55.4 | 50.9 | 0.12 | 61 | 49 | 0.001 | 45 | 40 | 0.07 | 63 | 53 | 0.09 |
| Smoking (%) | 43.2 | 62.8 | 0.001 | NR | NR | NR | 27 | 44 | 0.001 | NR | NR |  | 51.2 | 60.7 | 0.09 |
| Prior MI^c^ (%) | 18.2 | 10.5 | 0.02 | 10.9 | 7.9 | 0.061 | 19 | 12 | 0.004 | NR | NR |  | 9.9 | 19.9 | 0.03 |
| Prior HF^d^ (%) | NR | NR | NR | NR | NR | NR | 8 | 3 | 0.003 | 4 | 3 | 0.26 | NR | NR | NR |
| Prior stroke (%) | NR | NR | NR | 7.1 | 5.4 | 0.18 | 7 | 4 | 0.037 | 0.3 | 0.5 | 1 | NR | NR | NR |
| Prior PCI^e^ (%) | 13.6 | 7.6 | 0.04 | 8.5 | 8.8 | 0.8 | 15 | 10 | 0.008 | 6 | 6 | 0.51 | NR | NR | NR |
| Prior CABG^f^ (%) | NR | NR | NR | 3.1 | 1.5 | 0.03 | 2 | 2 | 0.92 | 7 | 5 | 0.11 | NR | NR | NR |
| Multivessel coronary disease (%) | 62.4 | 57.4 | NR | 50.8 | 43.7 | 0.026 | NR | NR |  | NR | NR |  | NR | NR | NR |
| Malignancy (%) | NR | NR | NR | 12.6 | 5.6 | <  0.001 | NR | NR | NR | 5 | 3 | 0.7 | NR | NR | NR |
| Chronic kidney disease (%) | 27.2 | 10.5 | <0.001 | 14.1 | 2.3 | <  0.001 | NR | NR | NR | 2 | 0.43 | 0.01 | NR | NR | NR |
| Glycoprotein IIb/IIIa inhibitors (%) | NR | NR | NR | NR | NR | NR | 15 | 15 | 0.95 | NR | NR | NR | 9.8 | 4.8 | 0.045 |
| Important exclusion criteria | Cardiogenic shock | | | patients who did not undergo PCI | | | use of fibrinolytic therapy, active serious infection | | | no information about the blood transfusion | | | none | | |
| Design | Retrospective | | | Retrospective | | | Prospective | | | Retrospective | | | Retrospective | | |
| Revascularisation with PPCI^g^ exclusive | No | | | No | | | Yes | | | no | | | yes | | |
| Mean follow-up (months) | 21 | | | 12 | | | 3 | | | 12 | | | 60 | | |

^a^*RBT* red blood cells transfusion, ^b^*NR* not reported, ^c^*MI* myocardial infarction, ^d^*HF* heart failure, ^e^*PCI* percutaneous coronary intervention, ^f^*CABG* coronary artery bypass graft, ^g^*PPCI* primary percutaneous coronary intervention

Supplementary Table 2. Quality assessment of the included studies using the Newcastle-Ottawa Scale (7)

| Study | Ergelen 2012 (7) | Gili 2016 (7) | Jolicoeur 2009 (7) | Shishehbor 2009 (7) | Tajstra 2013 (7) |
| --- | --- | --- | --- | --- | --- |
| Is the selected cohort representative? | Yes | Yes | Yes | Yes | Yes |
| Is the selection of controls appropriate? | Yes | Yes | Yes | Yes | Yes |
| Is the ascertainment of exposure appropriate? | Yes | Yes | Yes | Yes | Yes |
| Is the demonstration that outcome of interest was not present at the start of the study true? | Yes | Yes | Yes | Yes | Yes |
| Are the selected and control groups comparable concerning age/other controlled factors? | No/No | No/No | No/No | No/No | No/No |
| Is the independent or blind assessment stated in the paper? | No | No | Yes | No | No |
| Was follow-up long enough? | Yes | Yes | Yes | Yes | Yes |
| Was follow-up adequate? | Yes | Yes | Yes | Yes | Yes |
| Total number of stars | 6 | 6 | 7 | 6 | 6 |

Supplementary Table 3. The results of search through PubMed on the 2^nd^ of February 2017.

| Nr of search | Query | PubMed |
| --- | --- | --- |
| 1 | acute myocardial infarction AND transfusion | 665 |
| 2 | acute myocardial infarction AND red blood cell transfusion | 73 |
| 3 | STEMI AND transfusion | 163 |
| 4 | STEMI AND red blood cell transfusion | 18 |
| 5 | acute coronary syndrome AND transfusion | 301 |
| 6 | acute coronary syndrome AND red blood cell transfusion | 54 |
| 7 | coronary disease AND red blood cell transfusion | 238 |
| 8 | percutaneous coronary intervention AND red blood cell transfusion | 45 |
| 9 | angioplasty AND red blood cell transfusion | 27 |
|  | Total | 1584 |
